# Acute Pancreatitis Due to COVID-19 Active Infection

**DOI:** 10.7759/cureus.20410

**Published:** 2021-12-14

**Authors:** Frank H Annie, Julton Chumbe, Lauren Searls, Jessica Amos, James Campbell, Suzanne Kemper, Sarah Embrey, Muhammad Bashir

**Affiliations:** 1 Cardiology, Charleston Area Medical Center (CAMC), Charleston, USA; 2 Internal Medicine, Charleston Area Medical Center (CAMC), Charleston, USA; 3 Research, Charleston Area Medical Center (CAMC) Health Education and Research Institute, Charleston, USA; 4 Pharmacy, University of Charleston School of Pharmacy, Charleston, USA; 5 Gastroenterology, Charleston Area Medical Center (CAMC), Charleston, USA

**Keywords:** icu admission, inpatient admissions, mortality, covid-19, acute pancreatitis due to covid-19

## Abstract

Background

This study investigates the relationship between coronavirus disease 2019 (COVID-19) infection and acute pancreatitis. We present large registry data assessing the association between acute pancreatitis and mortality in patients with COVID-19 post-infection.

Methods

The researchers identified adult patients aged 18-90 years with COVID-19 infections in the TriNetX (COVID-19 research network) database between January 20, 2020, and June 1, 2021. The researchers identified n=1,039,688 cases divided into two cohorts: those with post-acute pancreatitis (n= 1,173) and those without post-acute pancreatitis (n=1,038,515) post COVID-19 infection having follow-up within a two-week period. The researchers then conducted a 1:1 propensity score match to account for differences in the cohorts and created two well-matched cohorts (n=1,173/1,173).

Results

Patients that developed acute pancreatitis had higher mortality (12.4% vs 3.7%, p<0.001), stroke (3.6% vs 1.7%, p=0.005), higher inpatient admissions (28.2% vs 10.6%, p<0.001), and higher rates of ICU admission (9.5% vs 3.2%, p<0.001).

Conclusion

In a large multinational federated database, we observed higher mortality, stroke, higher inpatient admissions, and higher rates of ICU admissions among patients with COVID-19 with pancreatitis.

## Introduction

The coronavirus disease 2019 (COVID-19) pandemic continues to be a global challenge. Information surrounding COVID-19 infection continues to grow, and its clinical manifestations are still not fully understood. Typically presenting with pulmonary symptoms, such as cough, shortness of breath, and fever, extrapulmonary manifestations are continuing to be recognized with multiple organ systems affected. Notably, gastrointestinal symptoms, including diarrhea, nausea, vomiting, and abdominal pain, are commonly seen [1]. Pancreatitis has also been identified with increasing incidence in COVID-19 infection [2], and in patients with COVID-19 infection, the development of pancreatitis is associated with increased length of stay and need for mechanical ventilation [3]. Incidence of acute pancreatitis among COVID-19 positive patients has been reported as 0.27% and 0.71% in two studies, however, many case reports and a few case series describe an association [2-3]. The relationship between COVID-19 infection and acute pancreatitis is varied in the literature and is often based on pancreatic enzyme levels alone [4]. A large, multicenter study has yet to be published. Utilizing data from the TriNetX clinical database, this paper presents the prevalence of acute pancreatitis development in patients with active COVID-19 infection and establishes the impact of acute pancreatitis as a prognostic indicator.

## Materials and methods

We used the TriNetX (COVID-19 research network) platform to assess COVID-19 patients with a lab-confirmed severe acute respiratory syndrome coronavirus 2 (SARS-CoV-2) diagnosis and confirmed acute pancreatitis diagnosis based upon International Classification of Diseases (ICD) codes.

We created additional sensitivity analyses and used a negative control of unrelated bleeding with no known relationship to the condition of acute pancreatitis. The researchers also constructed a 1:1 propensity match to control for literature-driven covariates for COVID-19, which included age, white, black, Hispanic, hypertension, chronic kidney disease, personal history of smoking, chronic obstructive pulmonary disease, alcohol dependence, and body mass index (BMI) <30.

Data source

The TriNetX Inc. (Cambridge, MA) database is a global federal research network that combines real-time data from electronic medical records. The platform combines international data sets into a user-friendly platform for data extraction in the form of ICD 10.

Study sample

The researchers queried TriNetX, which is a collection of 63 health care organizations from 10 countries, from January 20, 2020, to June 1, 2021. The researchers identified n=1,039,688 from the ages of 18-90 and used the COVID-19 infection (Table 1) and acute pancreatitis diagnosis codes within two weeks of the COVID-19 diagnosis with no previous diagnosis of pancreatitis in their medical records.

**Table 1:** List of Codes Used to Identify the Study Cohort Claim Evidence of COVID Infection

B34.2 – Coronavirus infection unspecified
B97.29 – Another coronavirus as the cause of diseases classified elsewhere
J12.81 – Pneumonia due to SARS-associated coronavirus
U07.1 – 2019 - nCoV acute respiratory disease (WHO) Laboratory Evidence of COVID Infection
94307-6 – SARS coronavirus 2N gene (Nucleic acid amplification using primer-probe set N1)
94308-4 – SARS coronavirus 2N gene (Nucleic acid amplification using primer-probe set N2)
94310-0 – SARS-like coronavirus N gene (presence) in an unspecified specimen by NAA with probe detection
94314-2 – SARS coronavirus 2 RdRp gene (presence) in an unspecified specimen by NAA with probe detection
94315-9 – SARS coronavirus 2 E gene (presence) in an unspecified specimen by NAA with probe detection
94316-7 – SARS coronavirus 2N gene (presence in an unspecified specimen by NAA with probe detection
B34.2 – Coronavirus infection unspecified
B97.29 – Another coronavirus as the cause of diseases classified elsewhere
J12.81 – Pneumonia due to SARS-associated coronavirus
Acute Pancreatitis
K85.9 – Acute pancreatitis
K85 – Acute pancreatitis
Excluded Codes
K85.1 – Biliary acute pancreatitis
K85.10 – Biliary acute pancreatitis without necrosis or infection
K85.11 – Biliary acute pancreatitis with uninfected necrosis
K85.12 – Biliary acute pancreatitis with infected necrosis
K91.89 – Other postprocedural complications and disorders of the digestive system
K86.01 – Exocrine pancreatic insufficiency
K85.20 – Alcohol-induced acute pancreatitis without necrosis or infection
K85.21 – Alcohol-induced acute pancreatitis with uninfected necrosis
K85.22 – Alcohol-induced acute pancreatitis with infected necrosis
K85.30 – Drug-induced acute pancreatitis without necrosis or infection
K85.31 – Drug-induced acute pancreatitis with uninfected necrosis
K85.32 – Drug-induced acute pancreatitis with infected necrosis

Exposure

The exposure will be those individuals who had a positive diagnosis of developing acute pancreatitis based on ICD 10 codes within two weeks post the initial diagnosis of COVID-19.

Statistical analyses

The TriNetX platform uses descriptive statistics as frequencies with percentages for categorical variables as mean ± standard deviation for continuous measures. The baseline characteristics were compared using Pearson’s chi-squared test for categorical variables. To account for possible differences in the cohorts, the researchers utilized a 1:1 propensity match to create two well-matched cohorts of n=1,173/1,173. The TriNetX platform uses a logistical regression to obtain listed propensity scores for each of the selected covariates as stated above. The logistic regression uses the Python libraries (NumPy and Sklearn). The propensity score platform also compares the final results to R to compare and verify them. The final step in the verification process uses a nearest neighbor function set to a tolerance level of 0.01 and a difference of value >0.1. Morality for the propensity score-matching cohort was determined using the Kaplan Meier method and the statistical difference between the differing risk factors and the differing measures of association for 550 days.

Sensitivity analyses

In order to understand if any outside variable or health outcomes could be affecting the outcomes of this study, a sensitivity analysis was performed looking at the endpoint of bleeding.

## Results

Overall, 1,039,688 patients were included in the study. The first cohort was 1,173 (0.01%) patients that had a confirmed case of acute pancreatitis within two weeks following a diagnosis of COVID-19, 96% of cases were located inside the United States and 4% outside of the United States. The second cohort of 1,038,515 cases that did not have a positive case of acute pancreatitis was 89% inside the United States and 11% outside the country. The acute pancreatitis cohort was more male (51.75% vs 44.79%, p<0.001) and had higher comorbidities. The prevalence of hypertension was 51.6% vs 25.2% (p<0.001), chronic kidney disease 21.1% vs 5.1% (p<0.001), personal history of smoking 14.7% vs 6.2% (p<0.001), heart failure 13.9% vs 4.2% (p<0.001) chronic obstructive pulmonary disease 10.2% vs 3.7% (p<0.001), and alcohol dependence 2.6% vs 0.62 (p<0.001) (Table 2).

**Table 2:** Baseline Characteristics (PSM Match) PSM: propensity score matching

Baseline Characteristic	Unmatched Cohorts				Propensity Matched Cohorts			
	Acute Pancreatitis (N=1,173)	No - Acute Pancreatitis (N=1,038,515)	P-Value	Standardized Mean Difference	Acute Pancreatitis (N=1,173)	No - Acute Pancreatitis (N=1,173)	P-Value	Standardized Mean Difference
Age at Index	55.4±16.7	47.9± 18.6	<0.01	0.43	55.4±16.7	55.9±17.1	0.48	0.03
White	57.80%	57.91%	0.94	0.02	57.80%	59.34%	0.50	0.03
Male	51.75%	44.79%	<0.01	0.14	51.75%	53.28%	0.46	0.03
Female	46.55%	54.68%	<0.01	0.16	46.55%	44.84%	0.40	0.03
Black or African American	21.57%	14.57%	<0.01	0.18	21.57%	20.21%	0.42	0.03
Hispanic or Latino	18.16%	12.30%	<0.01	0.16	18.16%	17.90%	0.87	0.01
Hypertensive diseases	51.58%	25.20%	<0.01	0.56	51.58%	51.83%	0.90	0.01
Chronic kidney disease (CKD)	21.14%	5.10%	<0.01	0.49	21.14%	20.89%	0.88	0.01
Smoking history	14.66%	6.22%	<0.01	0.27	14.66%	14.41%	0.87	0.01
Heart failure	13.90%	4.16%	<0.01	0.34	13.90%	13.90%	1.00	<0.01
Other chronic obstructive pulmonary disease	10.23%	3.71%	<0.01	0.26	10.23%	10.15%	0.95	0.03
Alcohol use	2.56%	0.63%	<0.01	0.15	2.56%	2.47%	0.90	0.01

**Figure 1:**
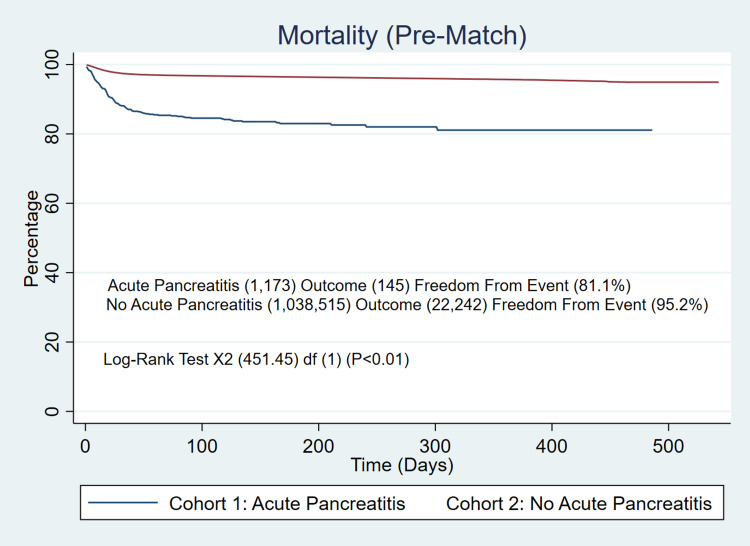
Pre-Matched Mortality

**Figure 2:**
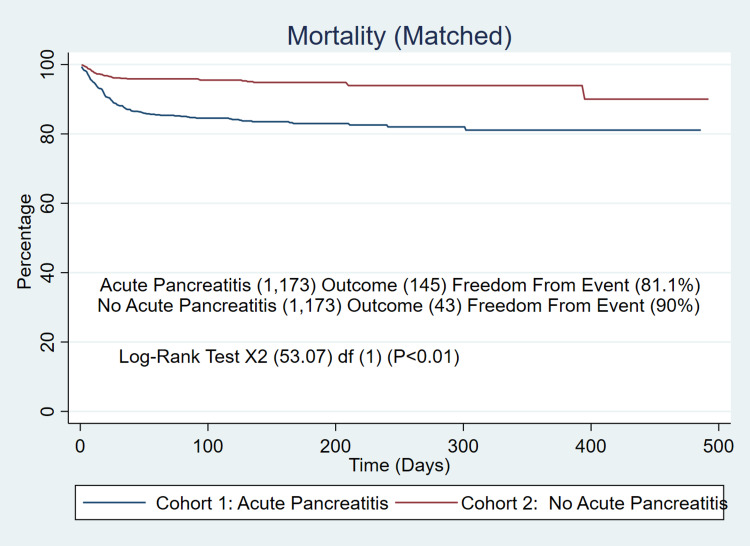
Matched Mortality

Before propensity score matching of the two cohorts, the all-cause mortality for the acute pancreatitis cohort was 12.3% (145/1,173) compared to the non-acute pancreatitis cohort, which was 2.1% (22,242/1,038,515). The additional log-rank test supported the initial findings (81% vs 95.2%, p<0.001) at 550 days (Figure 1). The post-propensity score-matched all-cause mortality additionally supported the initial findings, as well as the additional log-rank test (Figure 2), with a matched mortality difference of higher mortality of 12.4% vs 3.7% (p<0.001), cases of stroke (3.6% vs 1.7%, p=0.005), higher inpatient admissions (28.2% vs 10.6%, p<0.001), and higher rates of ICU admission of (9.5% vs 3.2%, p<0.001).

The researchers also conducted a falsification endpoint to account for any unmeasured confounders of bleeding, which had the result of 1.02% vs 0.9% (p=0.67).

## Discussion

Acute pancreatitis as a manifestation of COVID-19 infection has been reported in isolated case reports and case series, however, a large, national study has yet to be published [2,5-12]. One theory for pancreatitis development is through the binding of ACE2 receptors, which are present in pancreatic ductal, acinar, and islet cells [13-14].

Our understanding of the COVID-19 pandemic and pathogenesis of Sars-CoV-2 is dynamic and ever-evolving, with an increasing number of poor prognostic factors becoming recognized [15-16]. Acute pancreatitis (AP) development in a COVID-19 positive patient has been described to be associated with increased mortality, need for intensive care during hospitalization, length of stay (LOS), and need for mechanical ventilation [15-16]. Inamdar et al. describe an increase in the incidence of AP due to un-identified causes in COVID-19 positive patients compared to COVID-19 negative patients, implicating Sars-CoV-2 to be a possible causative agent [16]. Our study results are consistent with the development of AP in COVID-19 positive patients to have a poor prognostic indication.

An increase in all-cause mortality, ICU admission, and need for mechanical ventilation in the AP cohort of COVID-19 positive patients after propensity score matching indicates the development of AP can be considered a poor prognostic indicator in COVID-19 infection. The AP cohort also demonstrated a higher number of major comorbidities, including hypertension (HTN), personal smoking history, chronic kidney disease (CKD), heart failure, chronic obstructive pulmonary disease (COPD), and alcohol dependence. Our study represents a diverse patient population taken from a variety of ethnicities and geographic locations and additionally has a generous patient cohort of more than one million patients globally.

Our study is not without limitations. The determinant of acute pancreatitis in our study population was based on ICD-10 codes without the ability to distinguish if the diagnosis was based on serology alone or included CT evidence of acute pancreatitis and classic abdominal pain symptoms, as basing a diagnosis AP by serology alone in COVID-19 patients has been reported [4]. This study is unable to delineate if cases of AP in COVID-19 patients had other, common causes of AP such as gallstones, alcohol, etc., and we are unable to comment on SARS-CoV-2 as a pathogenic etiology for AP [16]. Our study is a retrospective chart review, having the inherited limitations of selection bias and inability to assess incidence.

## Conclusions

While is it has been shown that patients with COVID-19 can develop acute pancreatitis, the findings of this study indicate worse outcomes as illustrated with increased incidence of mortality, stroke, rates of inpatient admission, and higher rates of ICU admission. However, this cohort also had increased comorbidities. Further studies delineating these cofounders may help better establish risk assessment.
